# Supplementary material for: Association between oral microbiome and seven types of cancers in East Asian population: a two-sample Mendelian randomization analysis
Source: Front Mol Biosci. 2023 Nov 21;10:1327893. doi: 10.3389/fmolb.2023.1327893 (PMC10702768; doi:10.3389/fmolb.2023.1327893)
Supplement: Supplementary file 1 [file DataSheet1.ZIP › Supplementary Materials/MR plots for tongue/tongue═╝/Prostate cancer/pheno.1249_to_prostate cancer_leave_one_out.pdf]

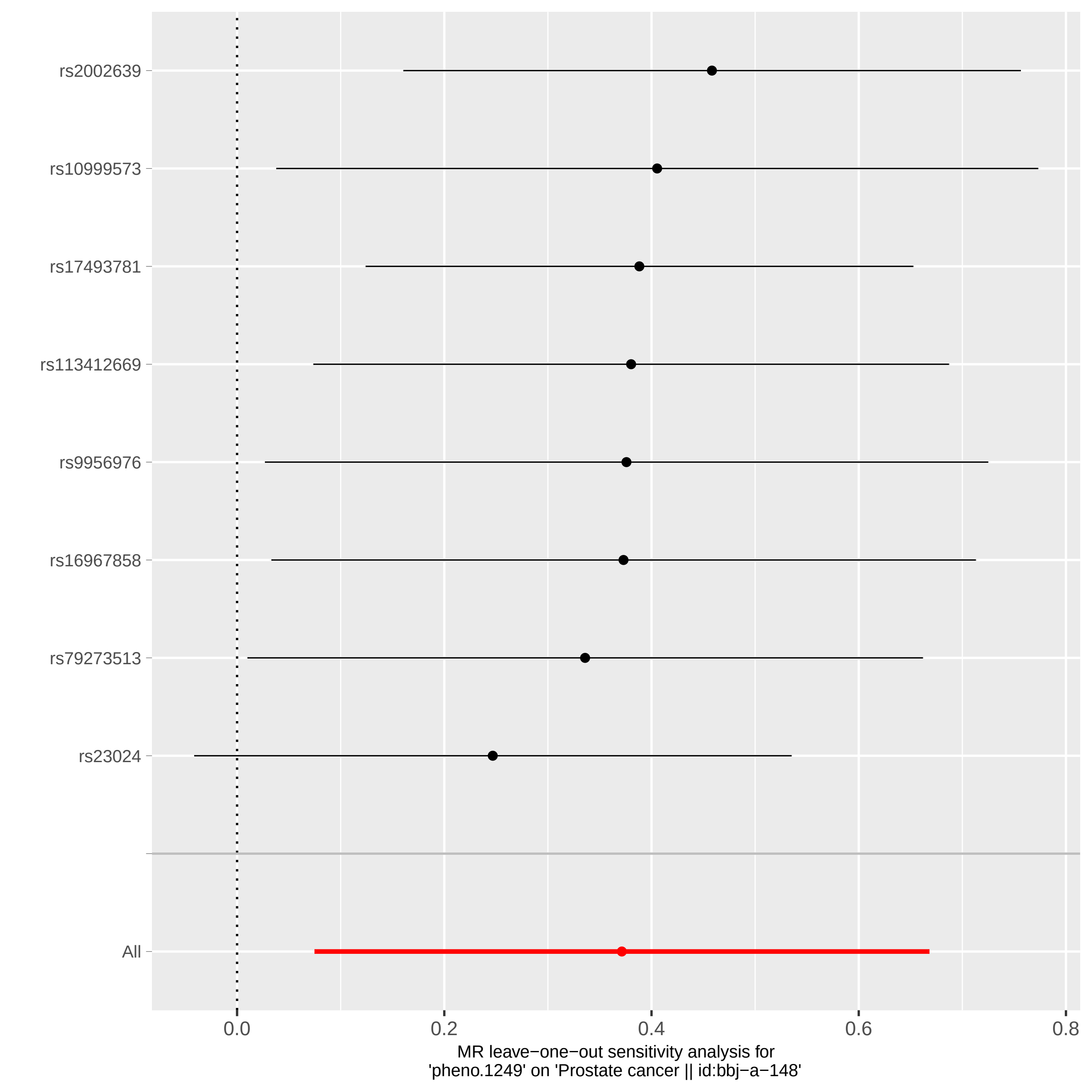

rs2002639
rs10999573
rs17493781
rs113412669
rs9956976
rs16967858
rs79273513
rs23024
All
0.0
0.2
0.4
0.6
0.8
MR leave−one−out sensitivity analysis for
'pheno.1249' on 'Prostate cancer || id:bbj−a−148'
